# Supplementary figures and images for: Protective Effect of l-Hexaguluroic Acid Hexasodium Salt on UVA-Induced Photo-Aging in HaCaT Cells
Source: Int J Mol Sci. 2020 Feb 11;21(4):1201. doi: 10.3390/ijms21041201 (PMC7072793; doi:10.3390/ijms21041201)

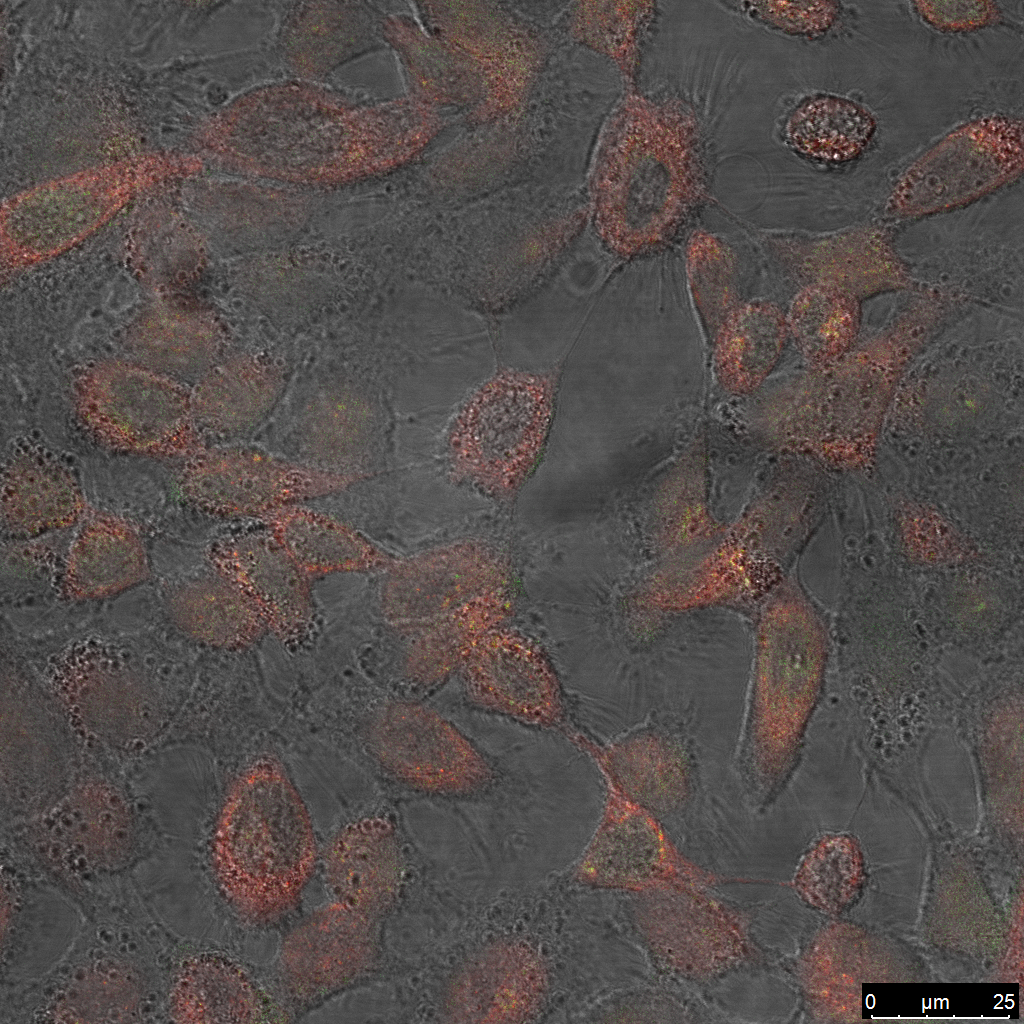

Supplement: Supplementary File 1 [file ijms-21-01201-s001.zip › 0.5h/LQ11.27_Series0.5H-G-1.tif]

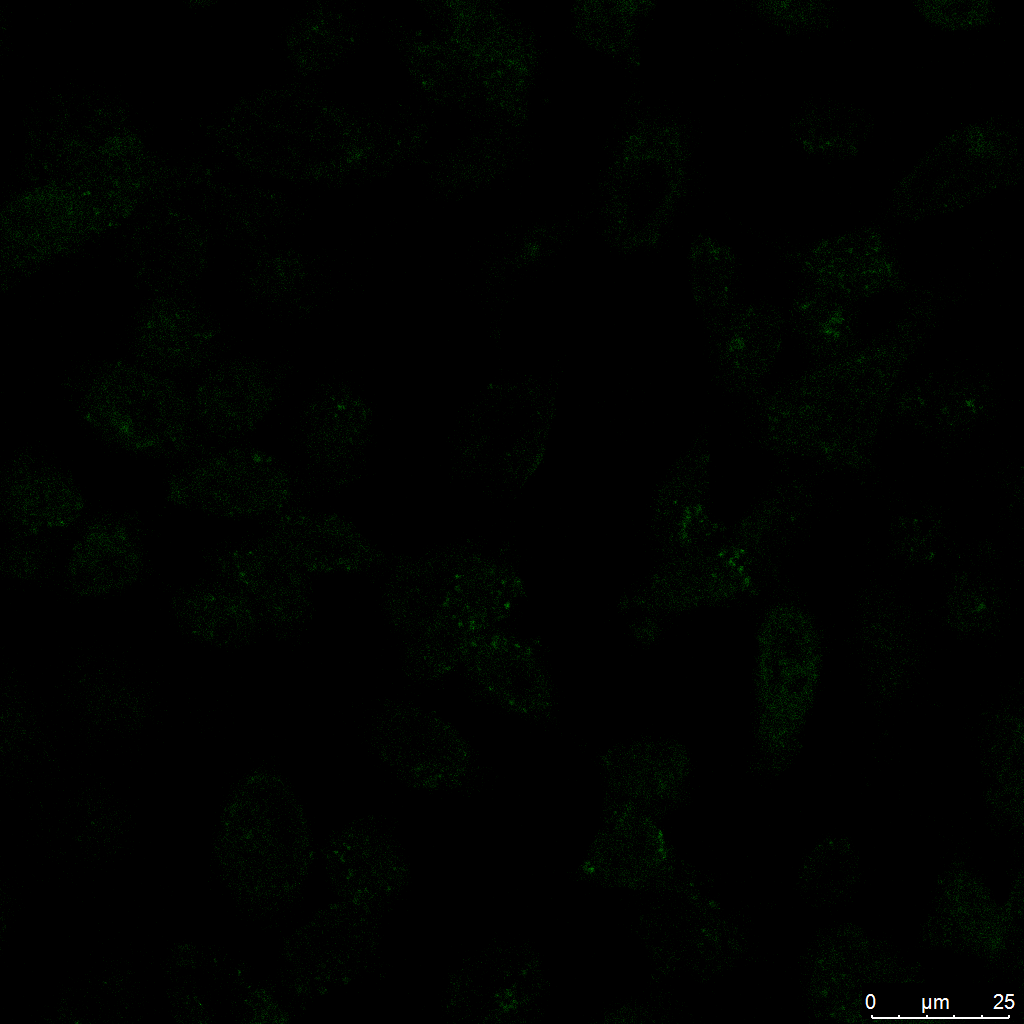

Supplement: Supplementary File 1 [file ijms-21-01201-s001.zip › 0.5h/LQ11.27_Series0.5H-G-1_ch00.tif]

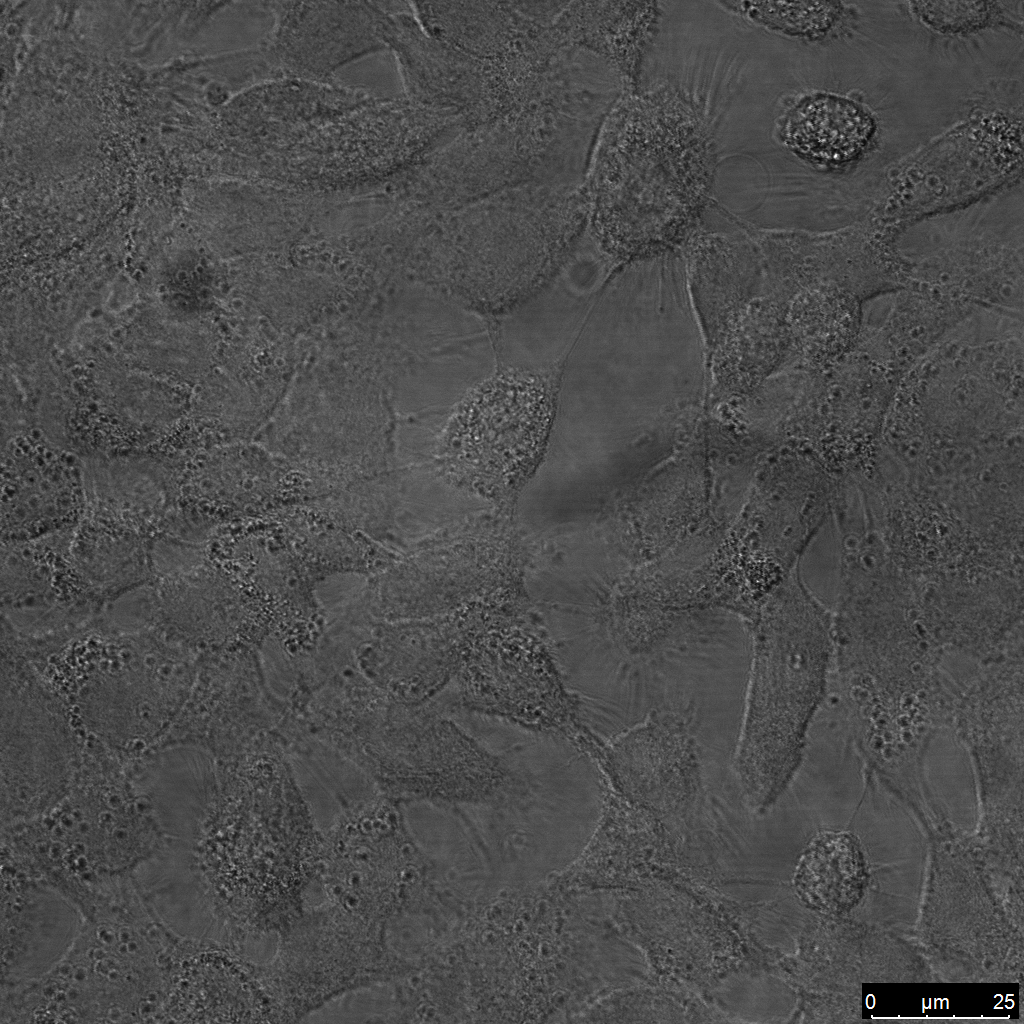

Supplement: Supplementary File 1 [file ijms-21-01201-s001.zip › 0.5h/LQ11.27_Series0.5H-G-1_ch01.tif]

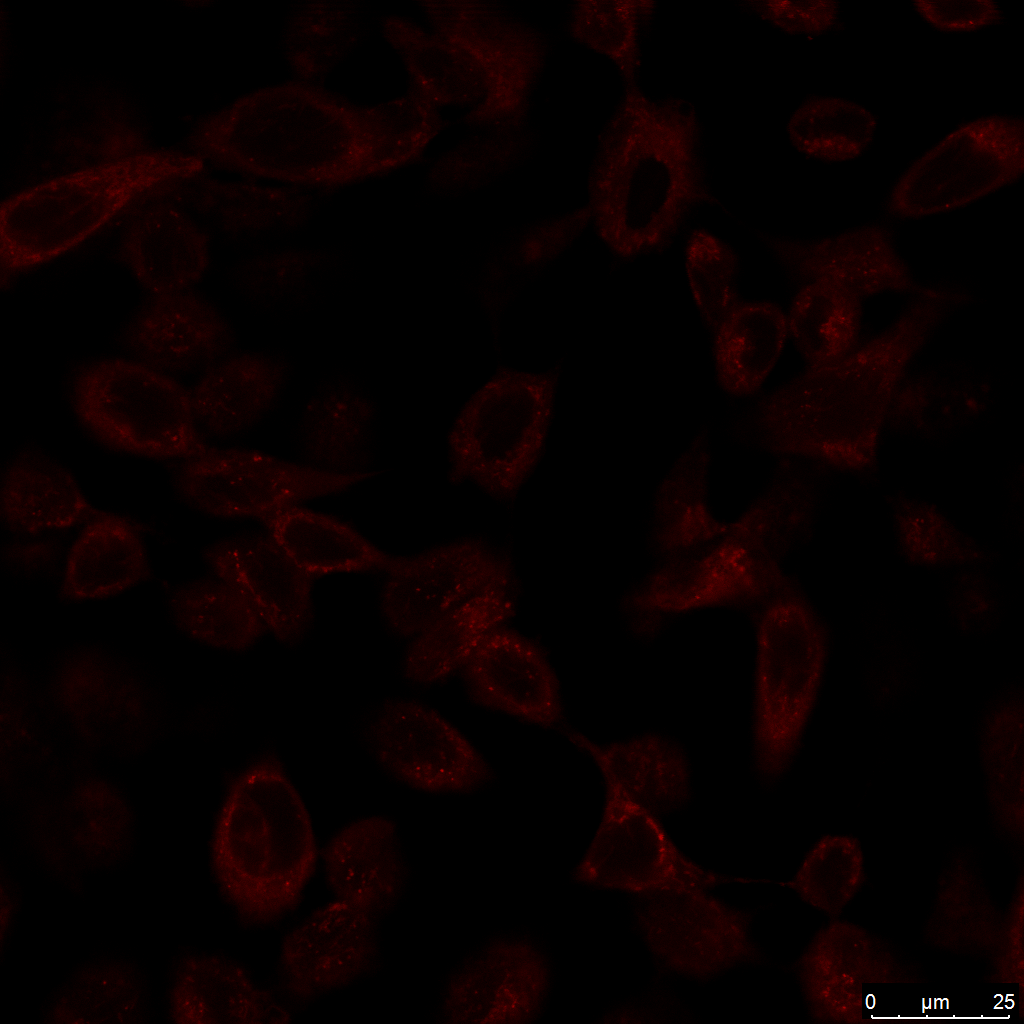

Supplement: Supplementary File 1 [file ijms-21-01201-s001.zip › 0.5h/LQ11.27_Series0.5H-G-1_ch02.tif]

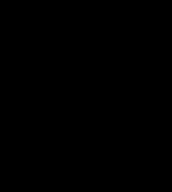

Supplement: Supplementary File 1 [file ijms-21-01201-s001.zip › 0H G/Project_Series003_ch00.tif]

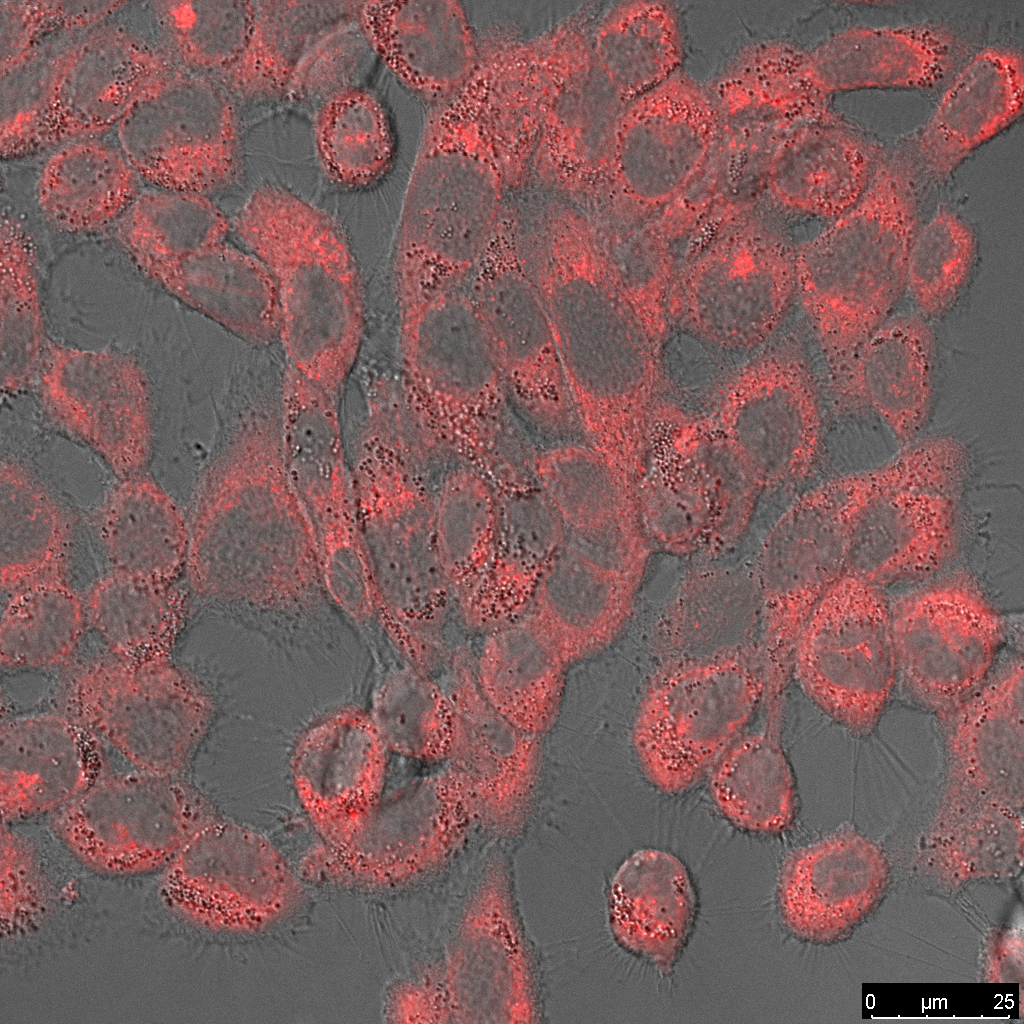

Supplement: Supplementary File 1 [file ijms-21-01201-s001.zip › 0H G/Project_Series034.tif]

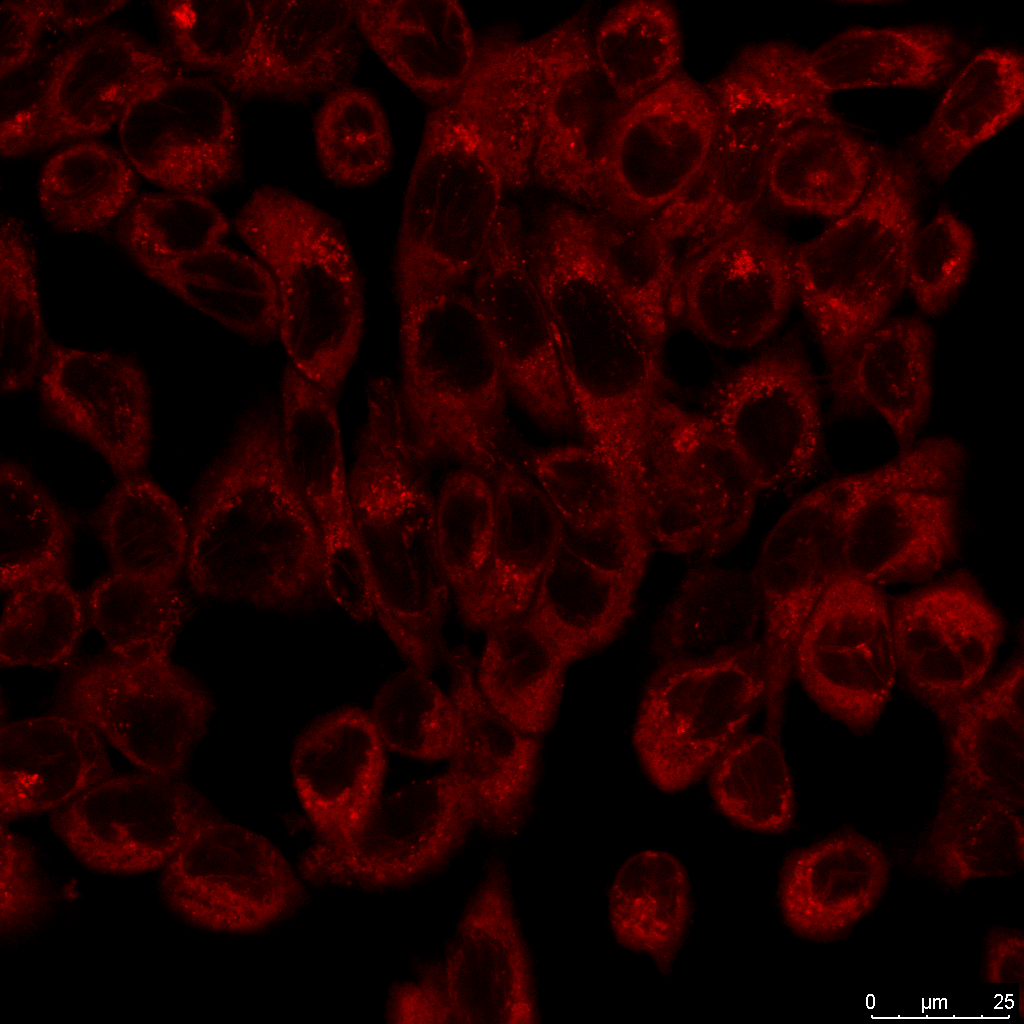

Supplement: Supplementary File 1 [file ijms-21-01201-s001.zip › 0H G/Project_Series034_ch00.tif]

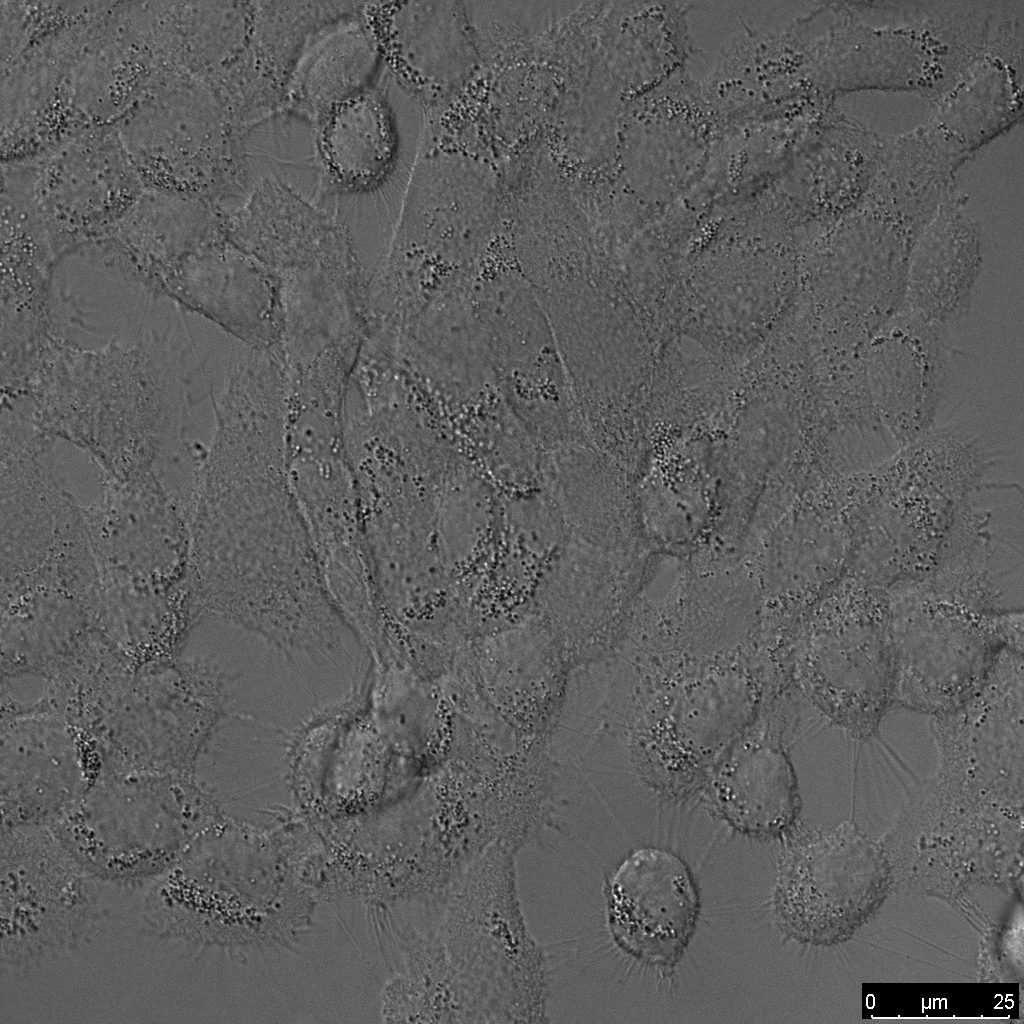

Supplement: Supplementary File 1 [file ijms-21-01201-s001.zip › 0H G/Project_Series034_ch01.tif]

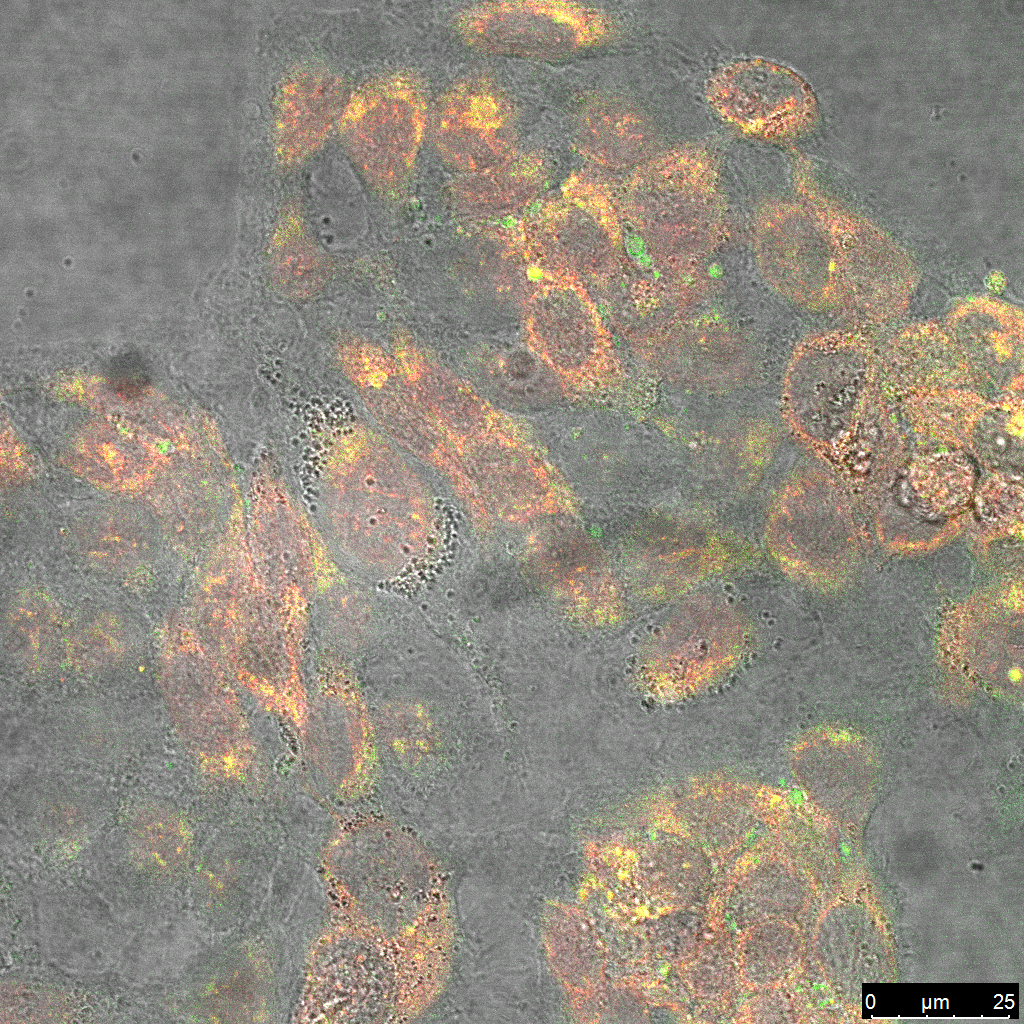

Supplement: Supplementary File 1 [file ijms-21-01201-s001.zip › 12H/LQ11.28_Series012H-G.tif]

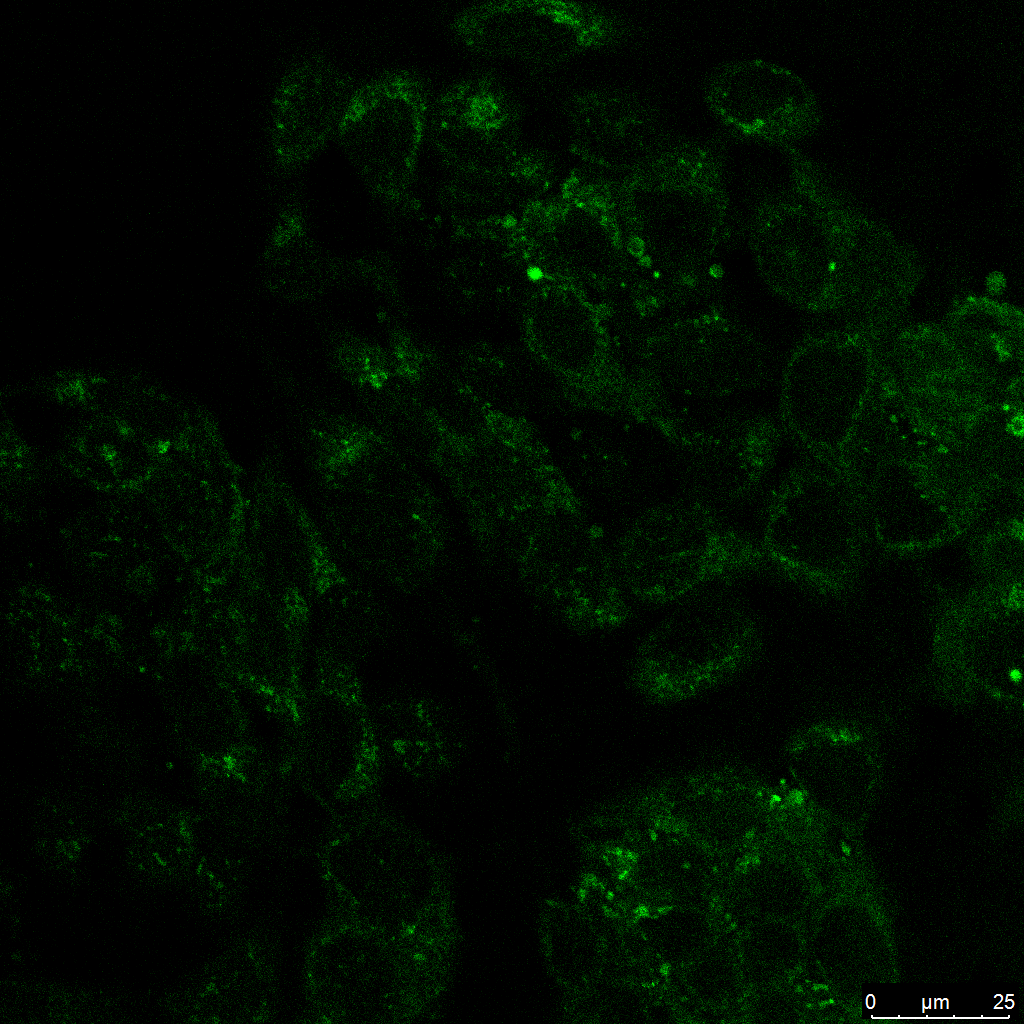

Supplement: Supplementary File 1 [file ijms-21-01201-s001.zip › 12H/LQ11.28_Series012H-G_ch00.tif]

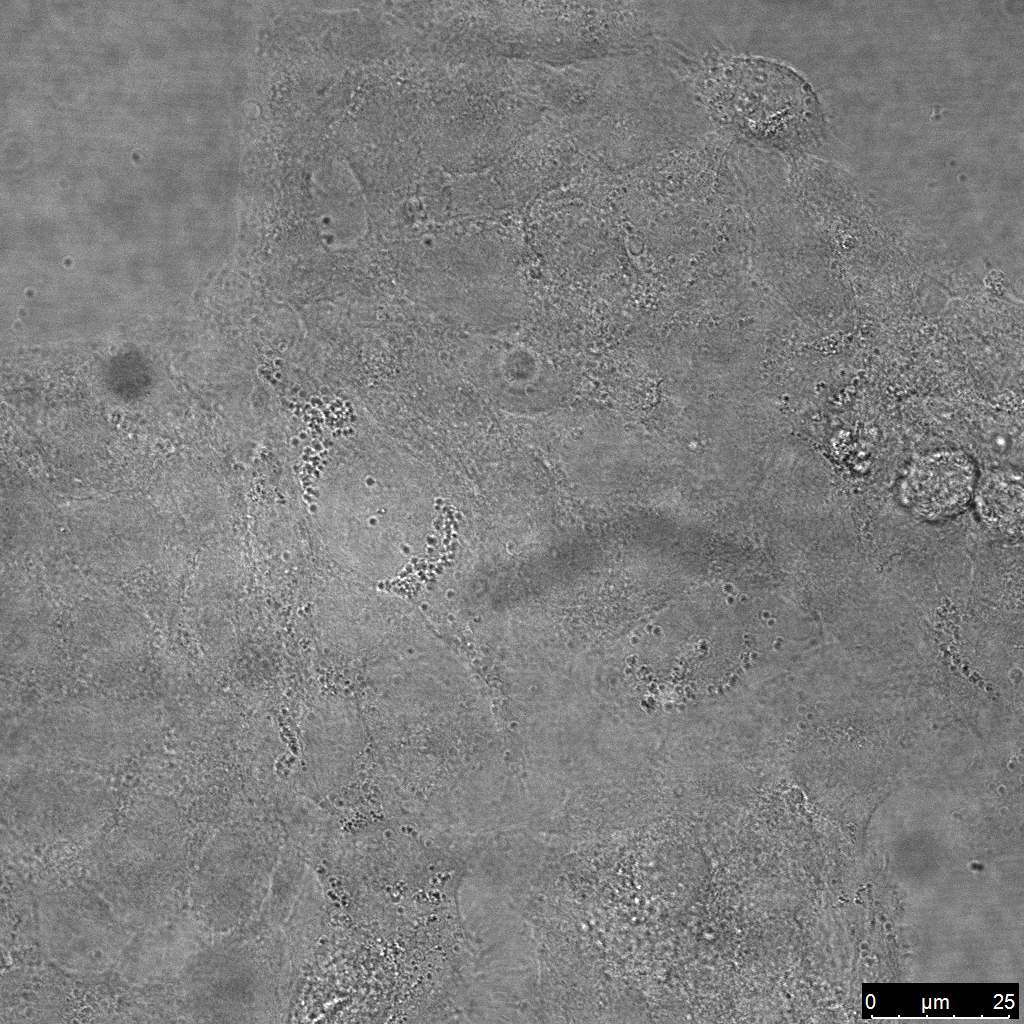

Supplement: Supplementary File 1 [file ijms-21-01201-s001.zip › 12H/LQ11.28_Series012H-G_ch01.tif]

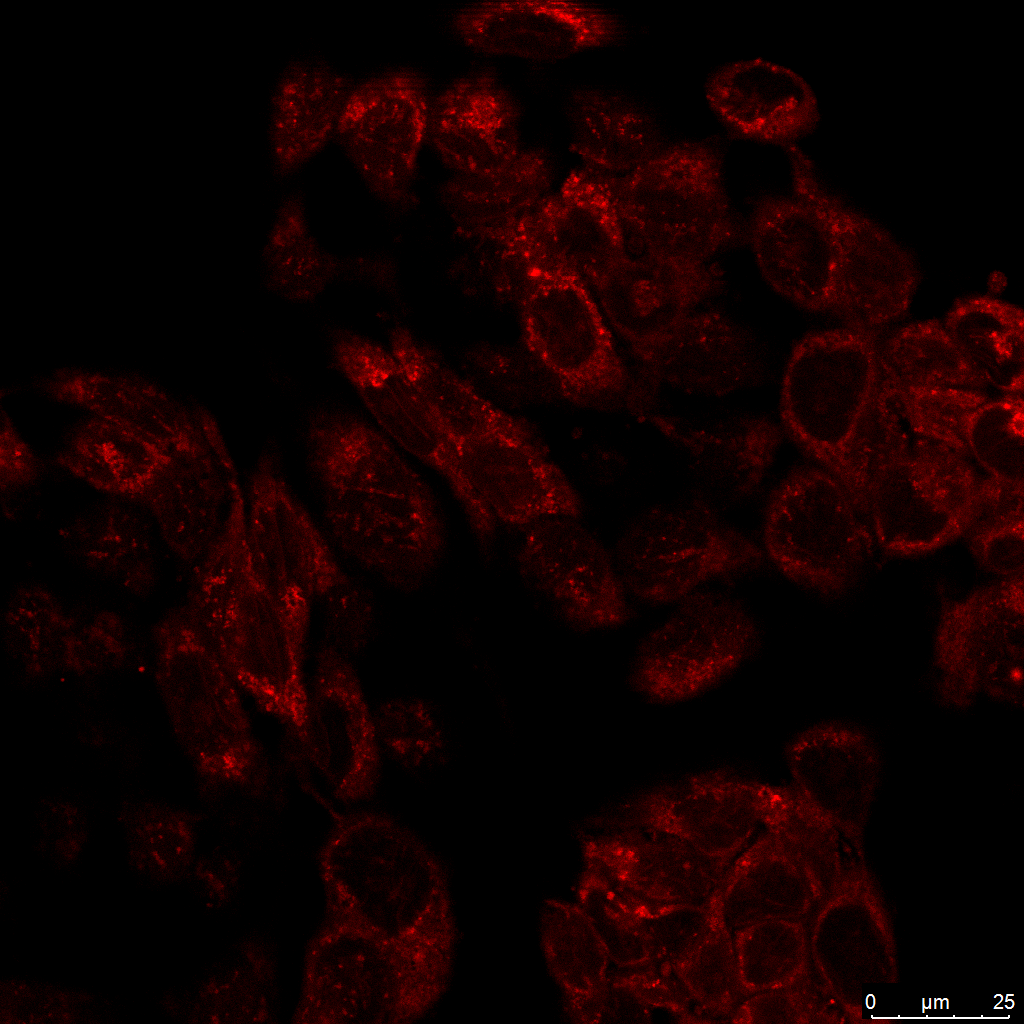

Supplement: Supplementary File 1 [file ijms-21-01201-s001.zip › 12H/LQ11.28_Series012H-G_ch02.tif]

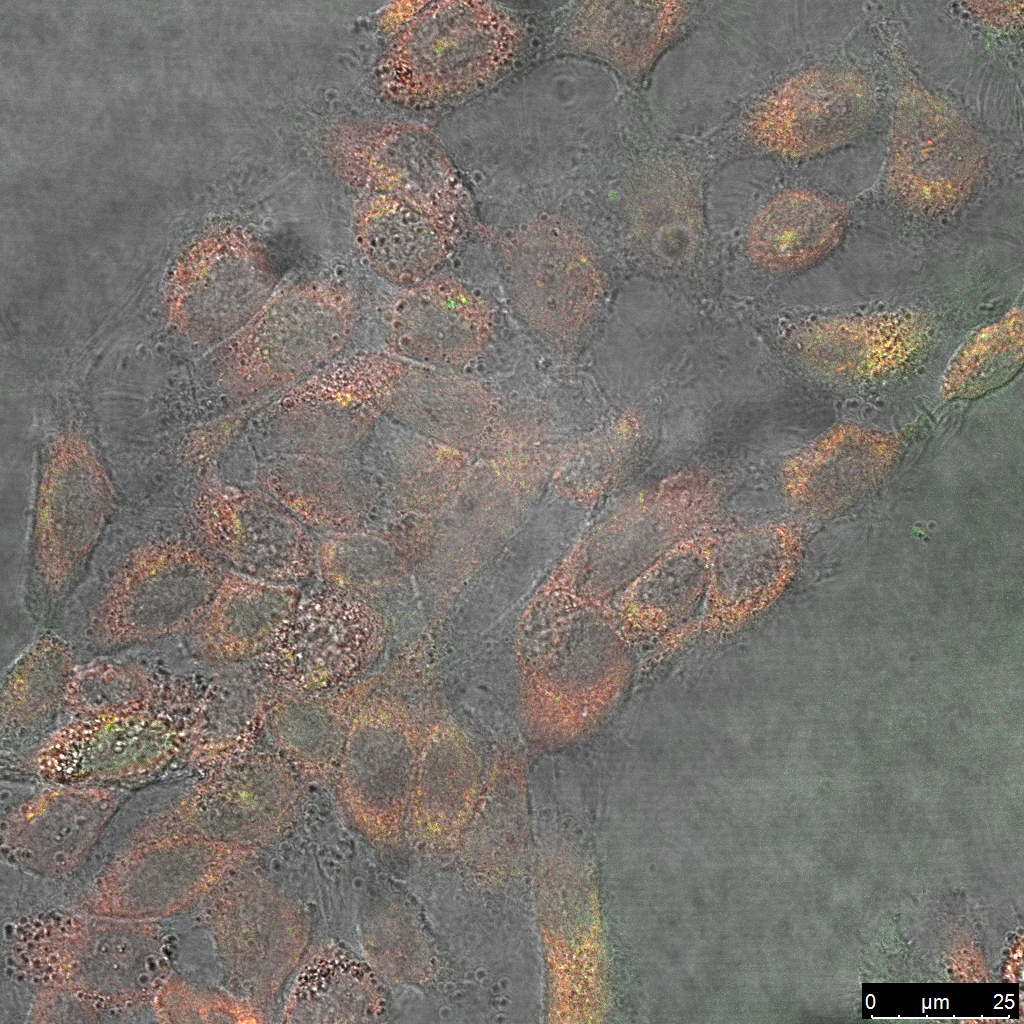

Supplement: Supplementary File 1 [file ijms-21-01201-s001.zip › 1H/LQ11.27_Series1H-G.tif]

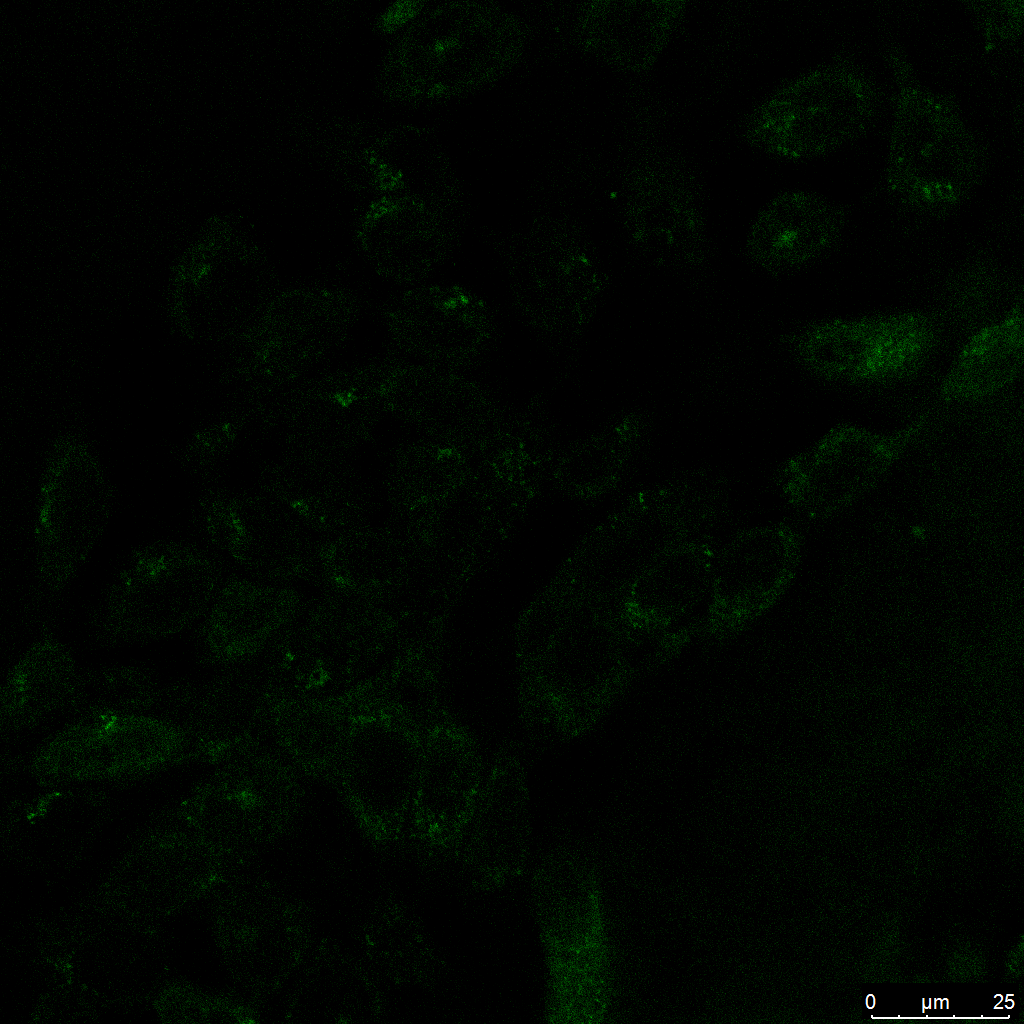

Supplement: Supplementary File 1 [file ijms-21-01201-s001.zip › 1H/LQ11.27_Series1H-G_ch00.tif]

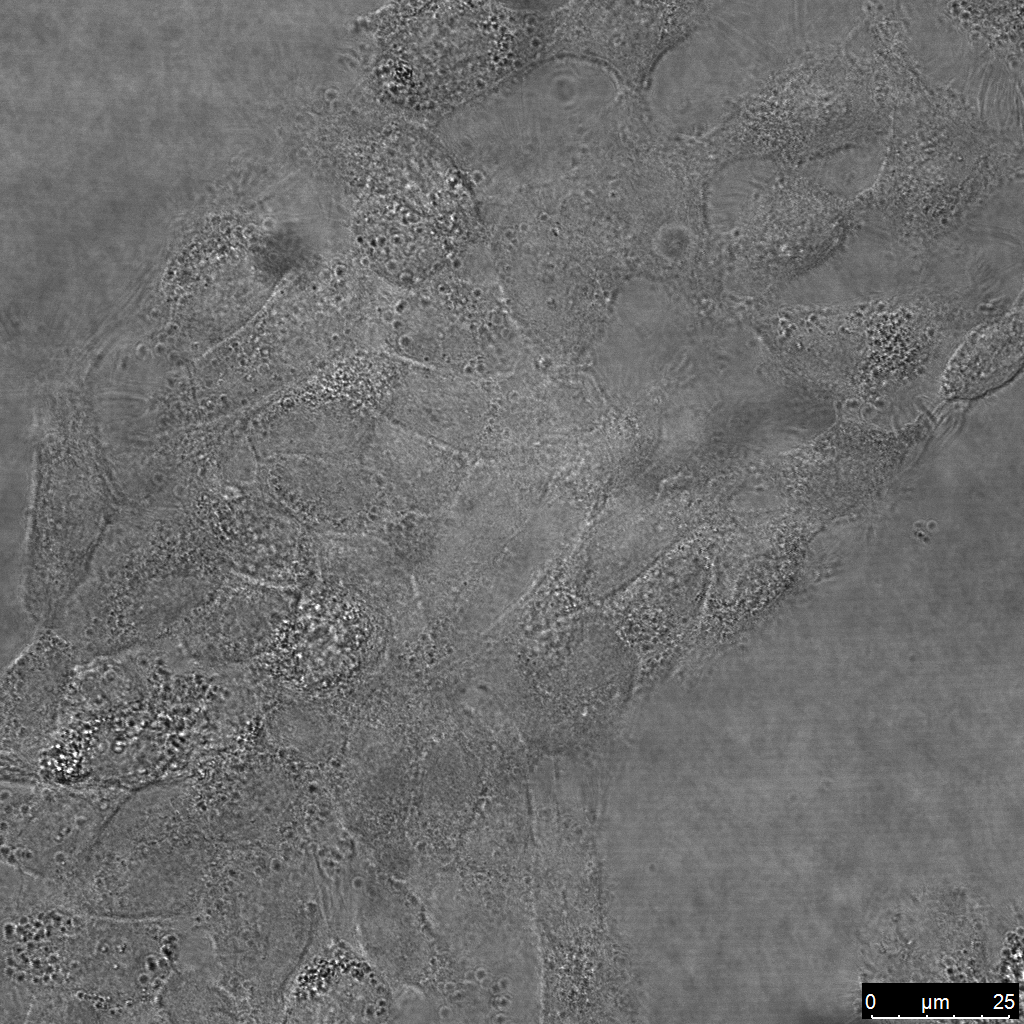

Supplement: Supplementary File 1 [file ijms-21-01201-s001.zip › 1H/LQ11.27_Series1H-G_ch01.tif]

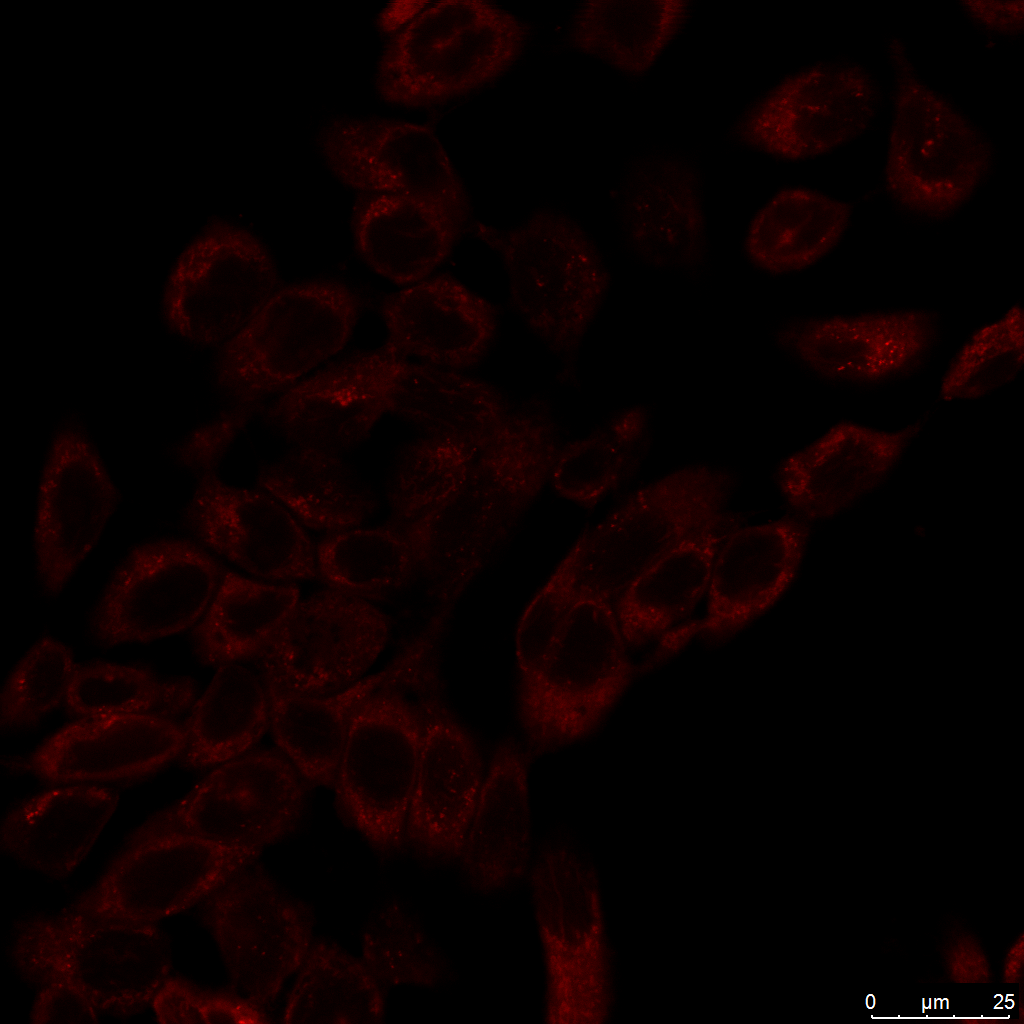

Supplement: Supplementary File 1 [file ijms-21-01201-s001.zip › 1H/LQ11.27_Series1H-G_ch02.tif]

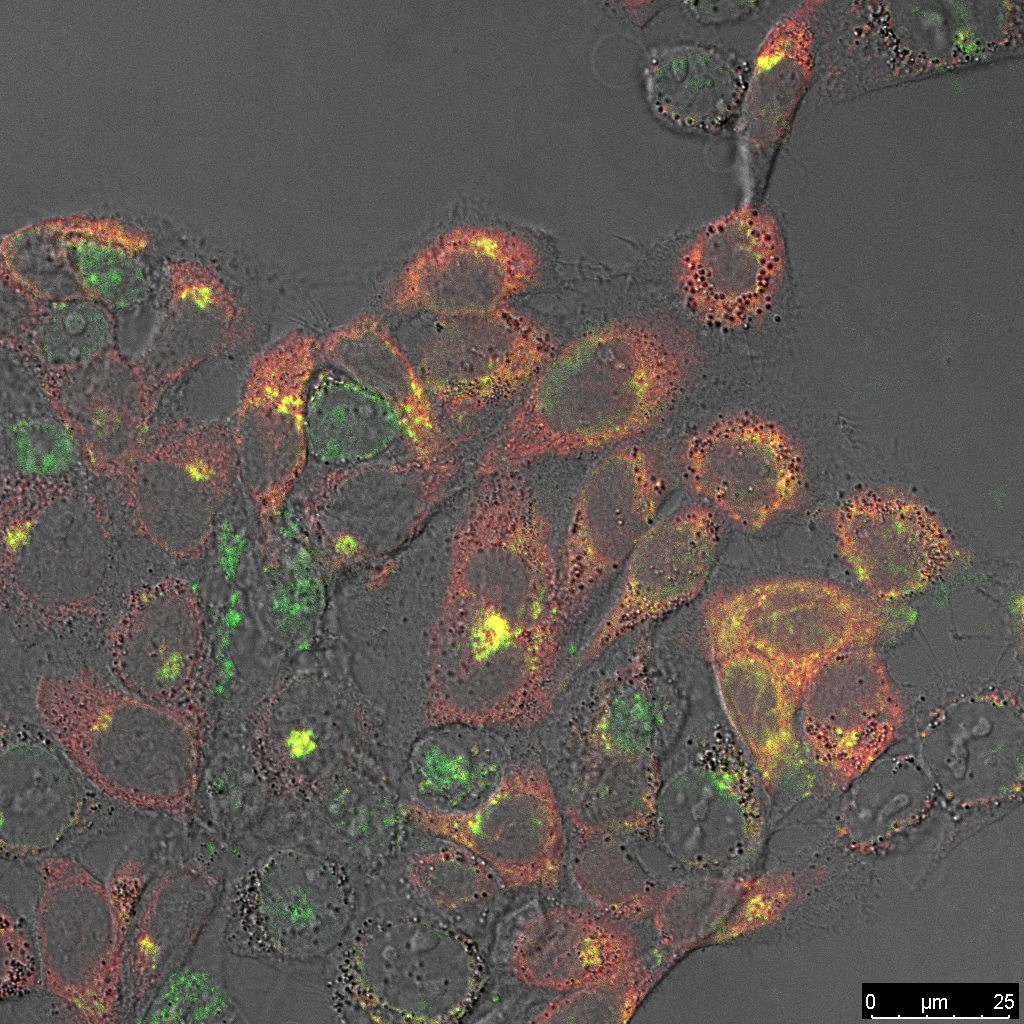

Supplement: Supplementary File 1 [file ijms-21-01201-s001.zip › 2H/Project_Series026.tif]

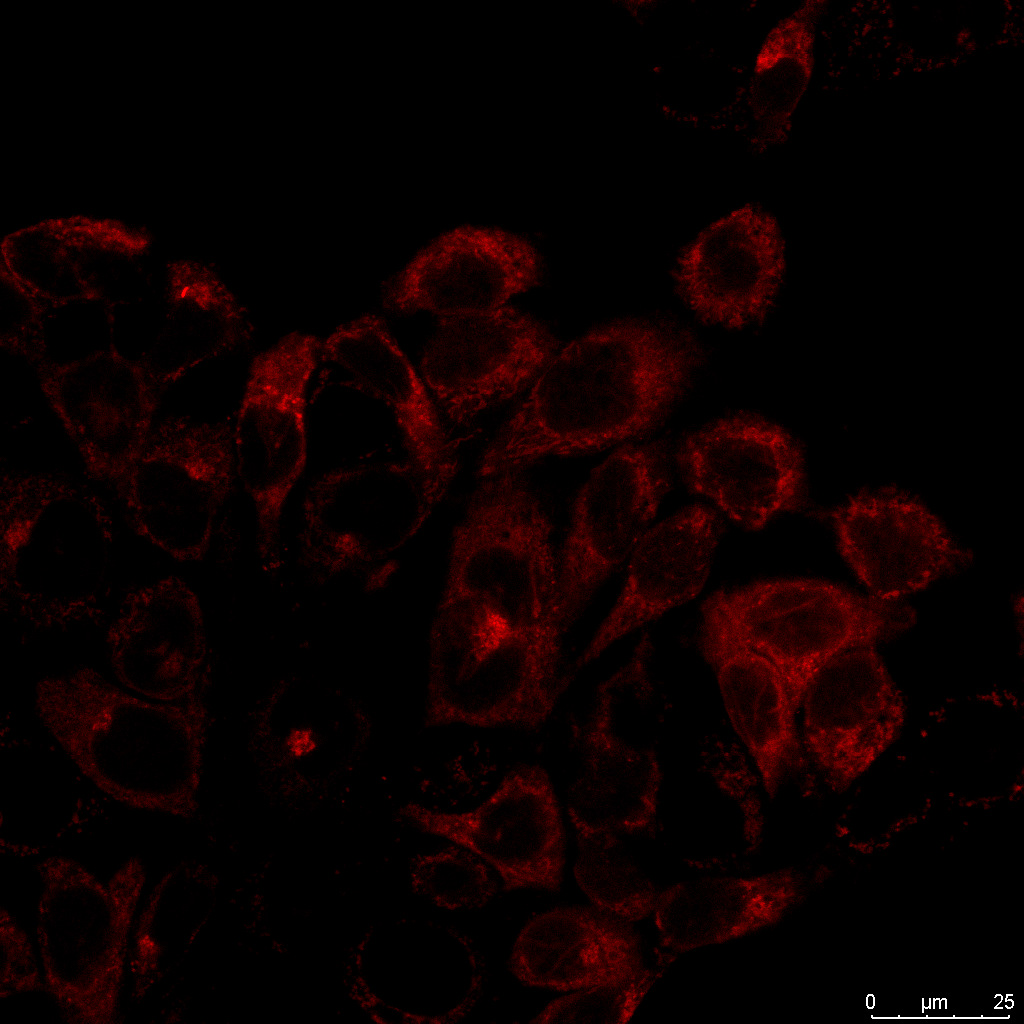

Supplement: Supplementary File 1 [file ijms-21-01201-s001.zip › 2H/Project_Series026_ch00.tif]

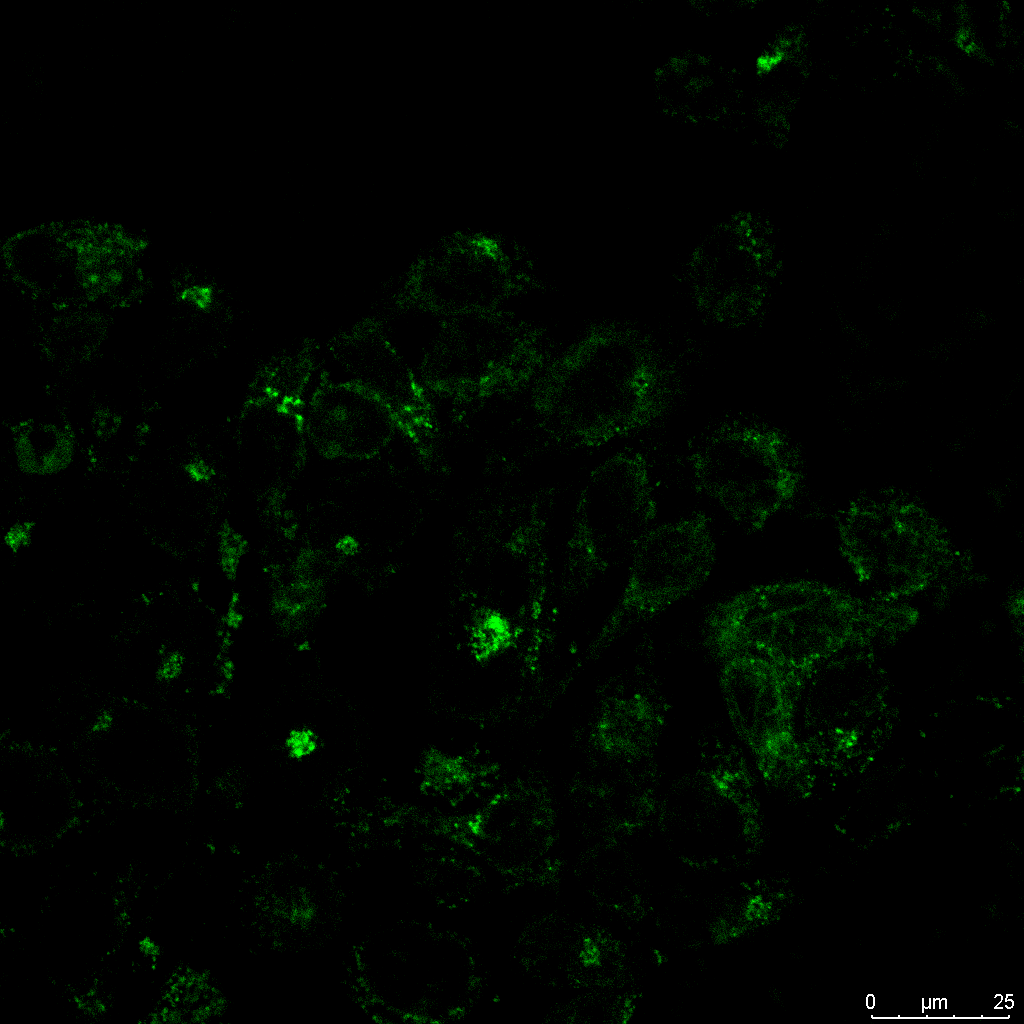

Supplement: Supplementary File 1 [file ijms-21-01201-s001.zip › 2H/Project_Series026_ch01.tif]

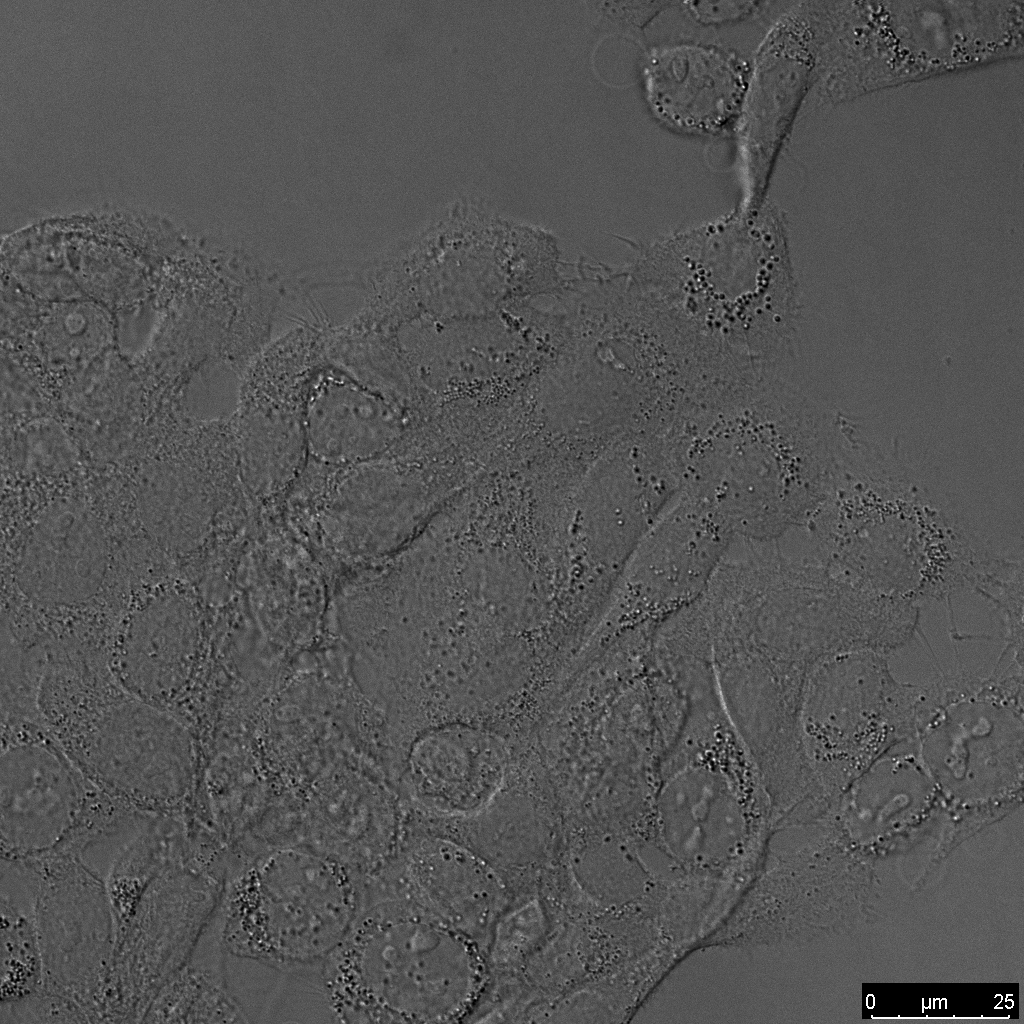

Supplement: Supplementary File 1 [file ijms-21-01201-s001.zip › 2H/Project_Series026_ch02.tif]

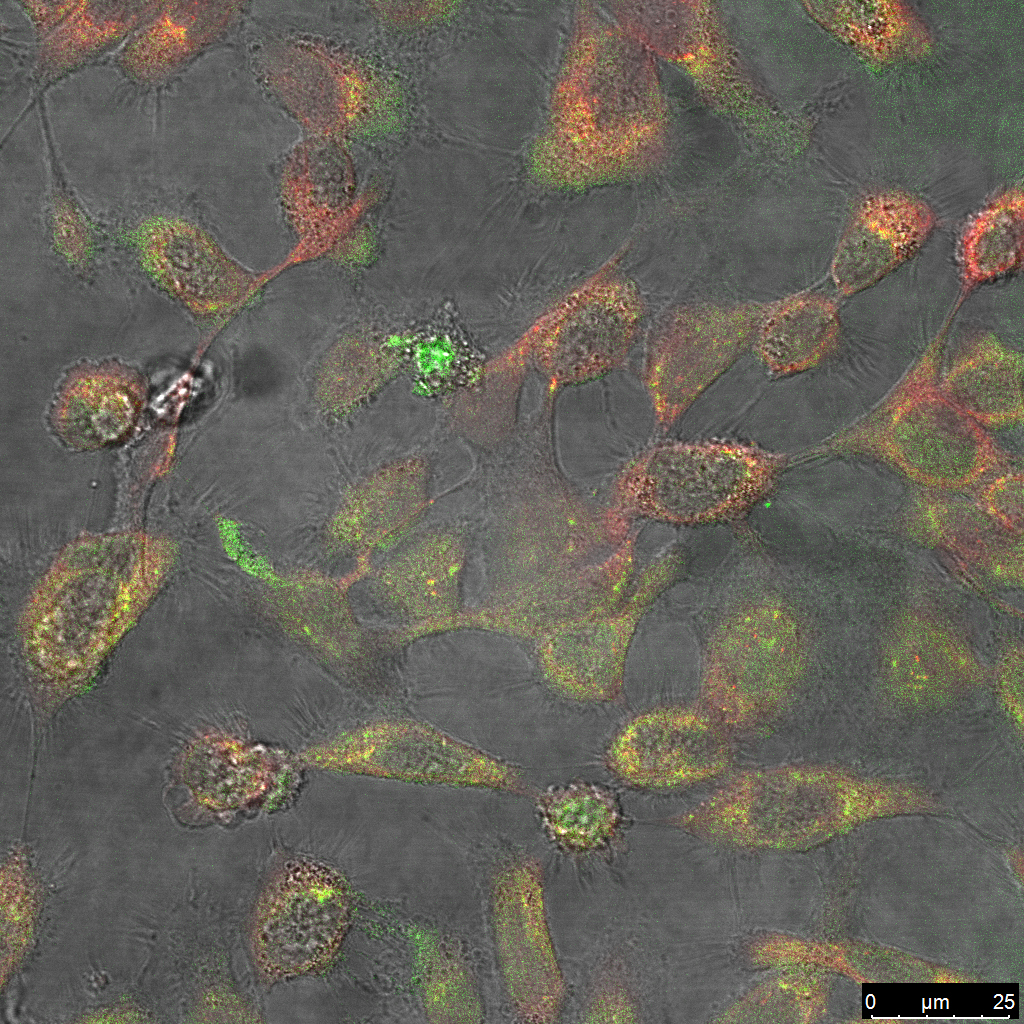

Supplement: Supplementary File 1 [file ijms-21-01201-s001.zip › 4H/LQ11.27_Series4H-G.tif]

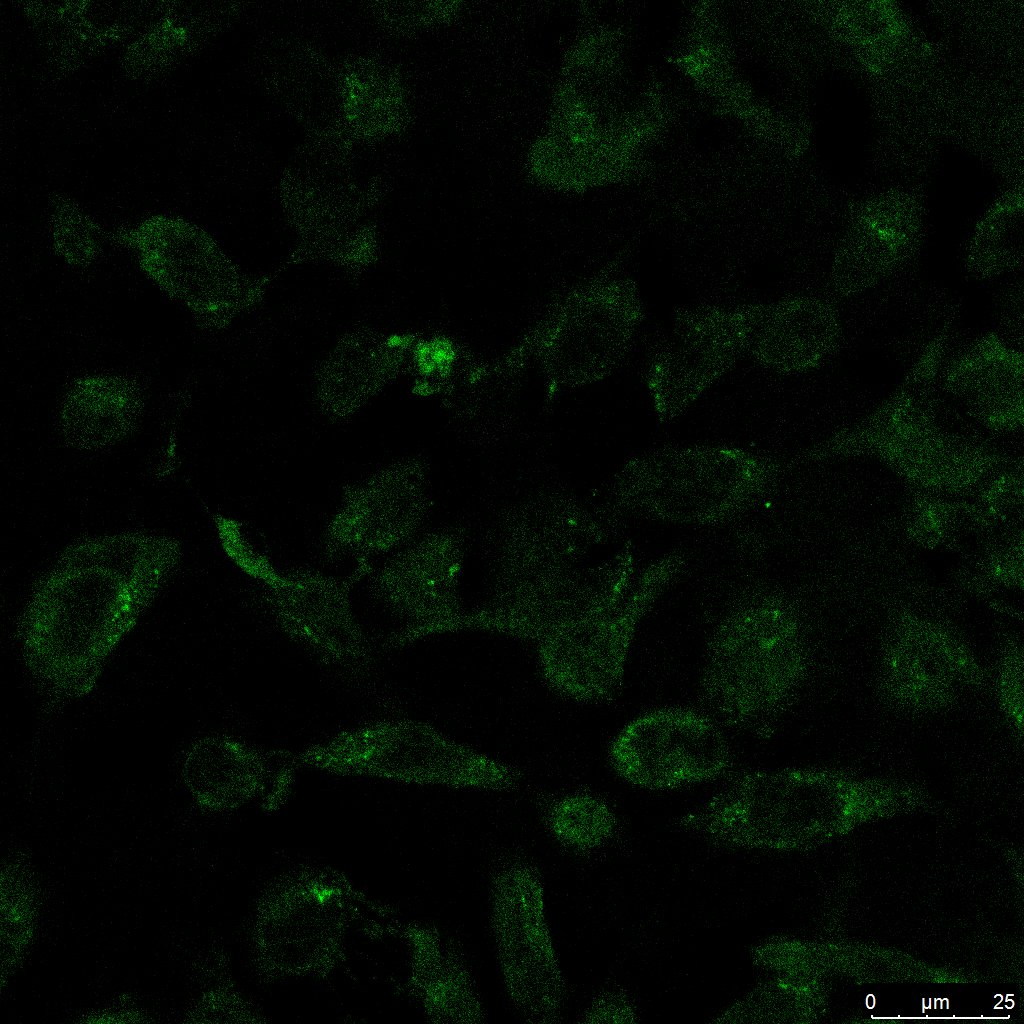

Supplement: Supplementary File 1 [file ijms-21-01201-s001.zip › 4H/LQ11.27_Series4H-G_ch00.tif]

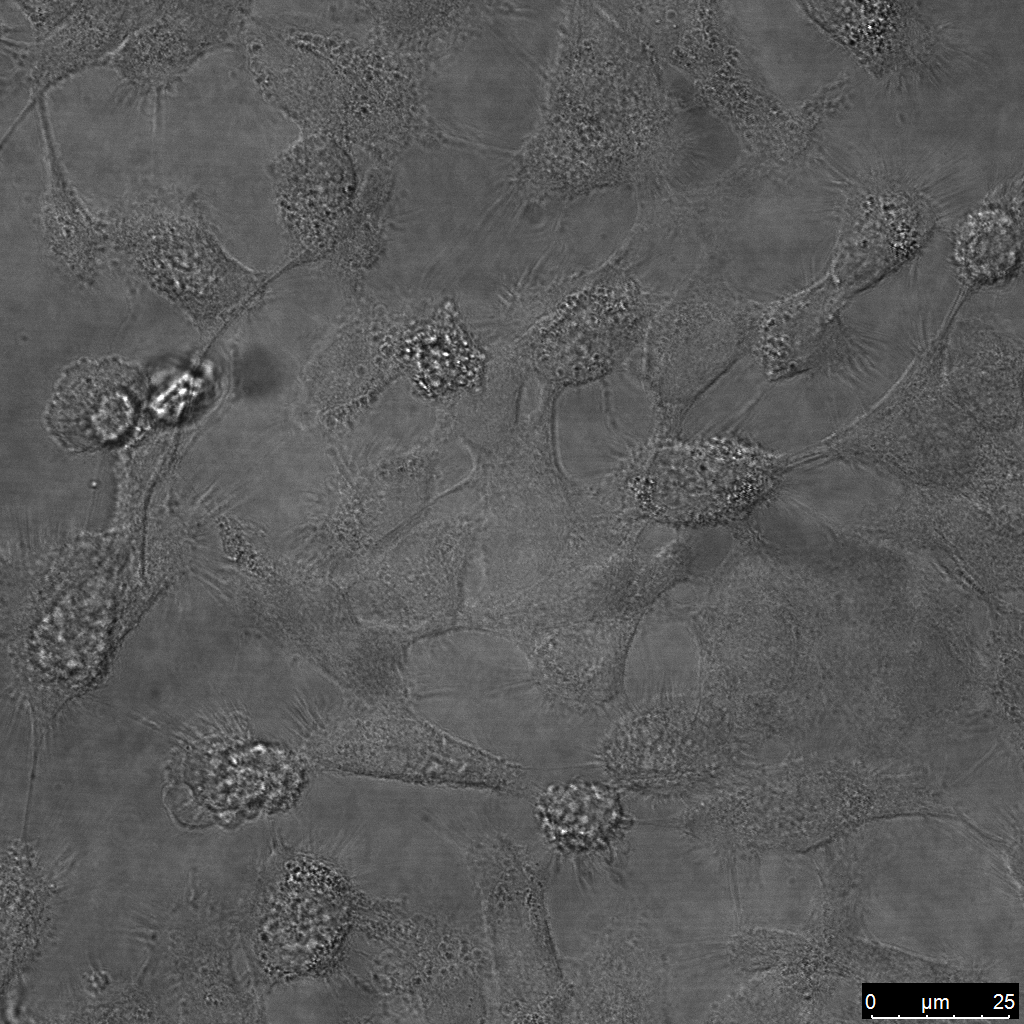

Supplement: Supplementary File 1 [file ijms-21-01201-s001.zip › 4H/LQ11.27_Series4H-G_ch01.tif]

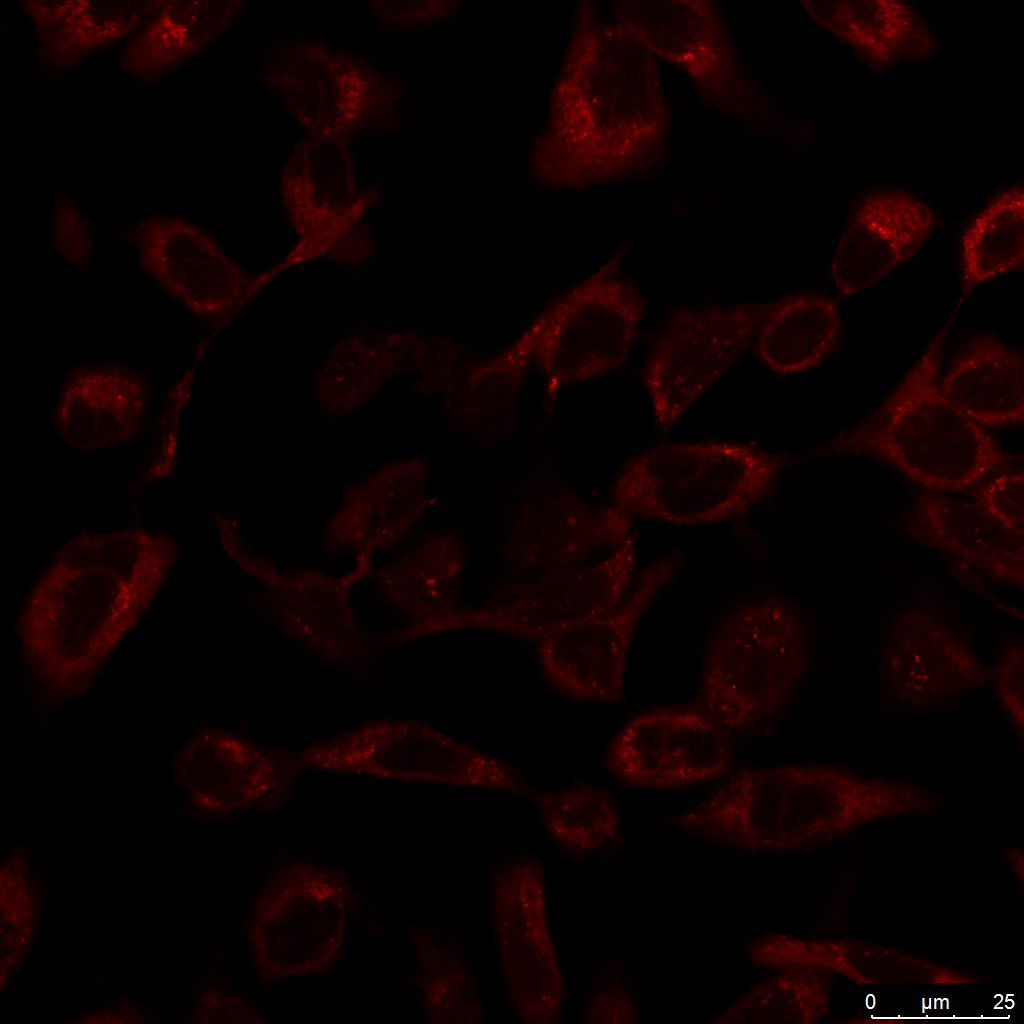

Supplement: Supplementary File 1 [file ijms-21-01201-s001.zip › 4H/LQ11.27_Series4H-G_ch02.tif]
